# Quantitative mapping of prefrontal oxygenation dysfunction and neuropsychiatric symptoms in patients with systemic lupus erythematosus: a multichannel functional near-infrared spectroscopy study

**DOI:** 10.1136/lupus-2026-002009

**Published:** 2026-07-01

**Authors:** Gengyi Chen, Chenxin Yu, Yifan Yang, Peng Ding, Ruotong Zhao, Ru Bai, Shuang Liu, Guofang Zhang, Shu Li, Xinyu Xu, Yuqi Cheng, Jian Xu

**Affiliations:** 1 Department of Rheumatology and Immunology, First Affiliated Hospital of Kunming Medical University, Kunming, China; 2 Neurology Department of the 920th Hospital of the Logistics Support Force of the People’s Liberation Army of China, Kunming, China; 3 Affiliated Mental Health Center, Zhejiang University School of Medicine, Hangzhou, China

**Keywords:** Lupus Erythematosus, Systemic, Information Science, Autoimmune Diseases

## Abstract

**Objective:**

Neuropsychiatric complications of systemic lupus erythematosus (SLE) lack relevant biological indicators that can be dynamically monitored. This study aims to break the technical barriers of functional near-infrared spectroscopy (fNIRS) in the detection of SLE brain function through methodological innovation and to provide data for establishing relevant biological indicators of neuropsychiatric symptoms in patients with SLE.

**Method:**

In this study, the dynamic data of oxyhaemoglobin (HbO) and deoxyhaemoglobin concentrations were obtained by using the spatiotemporal optimisation fNIRS paradigm (verbal fluency task, 60 s task period/10 s dynamic baseline correction) by integrating the verbal fluency task adapted to education and 37-channel fNIRS technology, and the activation effect of each channel in the prefrontal lobe was statistically analysed.

**Result:**

The concentration of HbO in the SLE group was significantly lower than that in the healthy controls (HCs) group (p<0.05) on 12 channels (CH13–15, 18, 21–25, 27, 28, 35). Compared with HCs, both the SLE with depression and SLE without depression subgroups showed significantly altered HbO concentrations across 14 channels (CH6, 9, 10, 13–15, 18, 24–28, 34, 36); however, no significant difference was detected between the two SLE subgroups. Compared with the HCs group and SLE without anxiety group, the SLE with anxiety group showed a more severe decrease in HbO concentration (p<0.05) on eight channels (CH6, 10, 13, 17, 18, 24, 25, 26), which were spatially concentrated in the dorsolateral prefrontal cortex and bilateral frontal pole region.

**Conclusion:**

Prefrontal HbO oscillation disorder is associated with depressive and anxiety symptoms in SLE patients, and its spatial gradient quantitatively maps the severity of these emotional symptoms during cognitive task engagement, providing a neuroimaging assessment tool that can be implemented at the bedside for neuropsychiatric SLE.

**WHAT IS ALREADY KNOWN ON THIS TOPIC**

- Neuropsychiatric systemic lupus erythematosus (NPSLE) affects 30%–40% of patients with SLE, with depression and anxiety being the most common manifestations.However, diagnosis remains highly dependent on clinical symptom assessment, lacking quantifiable dynamic relevant biological indicators that can be monitored at the bedside. Traditional neuroimaging techniques such as functional MRI and positron emission tomography are limited by poor temporal resolution, high cost or radiation exposure, making them unsuitable for rapid outpatient screening and longitudinal monitoring.

**WHAT THIS STUDY ADDS**

- This study establishes that prefrontal oxyhaemoglobin (HbO) oscillation dysfunction serves as a dynamic relevant biological indicators associated with depressive and anxiety symptoms in NPSLE during cognitive task activation. Using an education-adapted verbal fluency task with 37-channel fNIRS, we identified that patients with SLE with anxiety exhibit specific HbO reductions in the left dorsolateral prefrontal cortex and bilateral frontal pole (BA46 region), while depressive symptoms show a more distributed pattern across 14 channels, with no significant difference between depressed and non-depressed patients with SLE. These findings reveal symptom-specific spatial distribution patterns of prefrontal cortex dysfunction in SLE.

**HOW THIS STUDY MIGHT AFFECT RESEARCH, PRACTICE OR POLICY**

- This study provides a clinically feasible, non-invasive neuroimaging tool for bedside assessment of NPSLE, with potential applications as an auxiliary diagnostic aid, relevant biological indicators for treatment efficacy evaluation and predictor for identifying high-risk patients.
- The identification of BA46 as a key target region offers a neurobiological basis for developing targeted neuromodulation therapies such as transcranial magnetic stimulation.
- Future research should integrate fNIRS with immune-inflammatory markers and cerebrovascular parameters to further elucidate the pathophysiological mechanisms underlying neuropsychiatric symptoms in SLE.

## Introduction

 Systemic lupus erythematosus (SLE) is an autoimmune disease characterised by multiorgan involvement, with a high incidence of neuropsychiatric symptoms of SLE (NPSLE),^1 2^ and covering a variety of phenotypes such as cognitive impairment, mood abnormalities and psychotic symptoms, with depression and anxiety being the most common manifestations of diffuse central nervous system involvement in SLE. NPSLE significantly reduces the quality of life and increases mortality, and its diagnosis is still highly dependent on clinical symptom assessment,^3^ which lacks quantifiable dynamic relevant biological indicators support: on the one hand, the neuropathological mechanism of SLE involves multiple factors such as blood–brain barrier disruption, autoantibody deposition and microangiopathy,^4^,^6^ resulting in highly heterogeneous brain function damage. On the other hand, traditional neuroimaging techniques (such as functional MRI (fMRI) and positron emission tomography (PET)) are limited by spatiotemporal resolution mismatch (fMRI time resolution of about 0.5–2 s)^7 8^ or invasive risks (PET radioexposure),^9^ making it difficult to achieve rapid screening and long-term monitoring in outpatient settings.

NPSLE is a serious complication of SLE, with a variety of symptoms in the central, peripheral nervous system and psychiatric fields as the core, anxiety and depression are common phenotypes, NPSLE affects 30%–40% of SLE patients; among these, the prevalence of depression is 24%–39%,^10^ and this type of psychiatric symptoms can increase the risk of SLE activity, affect disease prognosis, and are also associated with higher morbidity and mortality. Although it is known that it may be related to neuroinflammation, blood-brain barrier disruption and autoantibody-mediated neuronal damage, the specific mechanism differences between these symptoms and other NPSLE phenotypes such as epilepsy and cerebrovascular events have not been clarified, and there is a lack of accurate biological markers that can be used for etiological identification and early diagnosis. In addition, there is a lack of evidence-based evidence for related treatment strategies, and the intervention plans for general anxiety and depression are mostly referenced, and the risk of drug interaction, treatment plans under different levels of activity, and the synergistic mode of psychology and drug treatment are all lacking in large-sample clinical trial verification, making it difficult to achieve personalised treatment. In summary, as a common severe phenotype of NPSLE, anxiety and depression have a heavy disease burden and extensive research gaps, highlighting the urgency of carrying out targeted research and improving the diagnosis and treatment system.

In recent years, functional near-infrared spectroscopy (fNIRS) technology has gradually become an emerging tool for brain function research due to its non-invasiveness, high temporal resolution (>10 Hz) and antimotion artefacts.^11 12^ fNIRS realises non-invasive, real-time, point-of-care monitoring of brain functional activity by detecting changes in haemoxidynamic changes in superficial cortical blood vessels,^13^ and its core principle is based on the characteristic absorption characteristics of haemoglobin to near-infrared light. This in turn reflects the neurological function status of this brain region. With the advantages of easy operation and moderate spatiotemporal resolution, this technology has preliminarily confirmed its potential to identify abnormal brain function in central nervous system diseases such as schizophrenia and depression.^14 15^ Studies have found that in patients with depression, fNIRS can detect asymmetry in the blood oxygen response of the prefrontal cortex, and this abnormal pattern is positively correlated with the severity of depression in patients.^16^ In patients with schizophrenia, this technology can capture the characteristic signal of decreased prefrontal-temporal cortex network synergy when performing cognitive tasks, providing a biological basis for the study of pathological mechanisms and auxiliary diagnosis of the disease.^17^ However, compared with its application in the field of mental illness, the research and application of fNIRS in the field of SLE, especially NPSLE, is still lacking.

As a classic paradigm for assessing executive function, the neural activation characteristics of the verbal fluency task (VFT) are closely related to the functional integration of the dorsolateral prefrontal cortex (DLPFC) and anterior cingulate gyrus,^18^,^20^ but previous SLE studies have obvious shortcomings, namely, ignoring the significant impact of education on language task performance, and failing to decouple the neural circuit overlap between emotional symptoms (depression/anxiety) and cognitive dysfunction. This makes it difficult for existing studies to distinguish whether abnormal prefrontal activation is due to the disease itself, or a derivative effect of differences in education level or psychiatric comorbidities. In subarachnoid haemorrhage, secondary metabolites of oxyhaemoglobin (such as heme) can destroy the BBB, aggravate cerebral oedema and oxygen supply insufficiency,^21^ and hypoxia can disrupt the endothelial cell connections of the BBB, exacerbating oxidative stress and mitochondrial dysfunction.^22 23^ As a ‘barrier’ to protect the brain, the blood–brain barrier can prevent harmful substances in the blood from entering the brain tissue and maintain the stability of the brain microenvironment. Mackay *et al*^24^ suggested that monitoring the change trend of blood oxygen concentration in the prefrontal cortex while completing cognitive tasks can reflect the cognitive level of the subjects. Therefore, this study intends to use oxyhaemoglobin as a marker of cognitive impairment and proposes a VFT paradigm and fNIRS fusion framework for educational adaptation, that is, to accurately regulate cognitive load through the three-level difficulty design of high-frequency words (primary school and below), medium-frequency words (middle school–preundergraduate), and low-frequency words (undergraduate and above), and at the same time integrate the Hamilton Anxiety/Depression Scale (HAMA/HAMD) and 37-channel fNIRS data to establish a symptom severity-brain activation quantitative response model. The study hypothesises that prefrontal blood oxygen oscillation disorders are dynamic relevant biological indicators associated with neuropsychiatric symptoms (depression and anxiety) in SLE and have symptom-specific spatial distribution patterns during cognitive task performance, and the validation of this hypothesis will provide living human brain evidence for the analysis of neural circuit associations between autoimmune disorders and neuropsychiatric symptoms, thereby promoting the early diagnosis of NPSLE and the development of targeted therapy.

## Materials and methods

A total of 77 patients with SLE who were admitted to the Department of Rheumatology and Immunology of the First Affiliated Hospital of Kunming Medical University and 54 healthy controls from June 2022 to December 2024 were enrolled.

### Inclusion criteria for SLE group

Patients aged 16–60 years who meet the 2019 EULAR/ACR classification criteria for SLE are assessed as right-handed by the Edinburgh Handed Inventory; no medication prior to fNIRS testing, or glucocorticoid maintenance dose (maximum permitted dose: prednisone-equivalent ≤10 mg/day) treatment was received, and the dose remained stable for ≥2 weeks. Hydroxychloroquine and immunosuppressants (including mycophenolate mofetil, tacrolimus, cyclosporine, methotrexate, leflunomide) were treated for at least 8 weeks with stable doses maintained for ≥4 weeks. Female subjects are non-pregnant and non-lactating; all subjects voluntarily participate in this study have been fully informed and signed a written informed consent form and can cooperate with the completion of MRI examination and related questionnaire assessments.

### Exclusion criteria for SLE group

Those who are combined with organic encephalopathy or neurological diseases that can affect brain structure are excluded, including patients with a history of head trauma or surgery, Parkinson’s disease, epilepsy, stroke, etc; those who have been diagnosed with severe mental illnesses such as depression, anxiety and schizophrenia in the past are excluded; those with a history of alcohol abuse, drug abuse or who have been treated with antidepressants or antipsychotics are excluded; those with other autoimmune diseases are excluded; those who are combined with severe hypertension, diabetes, renal insufficiency and other serious physical diseases that may affect brain structure are excluded. Subjects who have received the following treatments within a specific time window prior to enrolment are also excluded: plasmapheresis <6 months, B-cell depleting therapies such as rituximab, otuzumab and orezolizumab <1 year, belimumab, aflibercept, telitacicept, anti-TNF preparations and other biologics with immunosuppressive effects may interfere with the assessment of SLE disease activity <3 months, intravenous human immunoglobulin, granulocyte colony-stimulating factor <1 month, cyclophosphamide <1 month, lenalidomide, thalidomide <1 month, strong opioids <1 week, investigational drug or drugs not mentioned above <5 half-lives; those who cannot cooperate with the completion of the scale assessment and questionnaire filling are excluded.

### Inclusion criteria for healthy controls

Healthy participants who match the age, gender and education level of the patient group and are judged to be right-handed according to the Edinburgh Hand Advantageous Scale; no previous serious physical or mental illness, physical and mental health, non-lactating or pregnant women; a comprehensive physical examination of the enrolled subjects was performed by an experienced rheumatologist and a neurological examination by an experienced psychiatrist, and screening was performed using the American Diagnostic and Statistical Manual of Mental Disorders Fourth Edition (DSM-IV) Non-Patient Version Structured Clinical Interview (SCID-NP) method, and the results were normal. Those with normal T1 and T2-weighted imaging scans of conventional head MRI. all study participants voluntarily participated in this study and signed the informed consent form and were able to cooperate with the fNIRS examination and questionnaire evaluation.

### Psychological Assessment Scale

Depression and anxiety were assessed by the same psychiatrist on the day of the MRI examination using the Hamilton Depression Scale (HAMD) and the HAMA. The score of disease activity and the psychological assessment scale were performed within 2 days before or after the fNIRS examination. HAMD≥8 was divided into depression group, and HAMA≥7 was divided into anxiety group.

### fNIRS data acquisition

**Figure 1:**
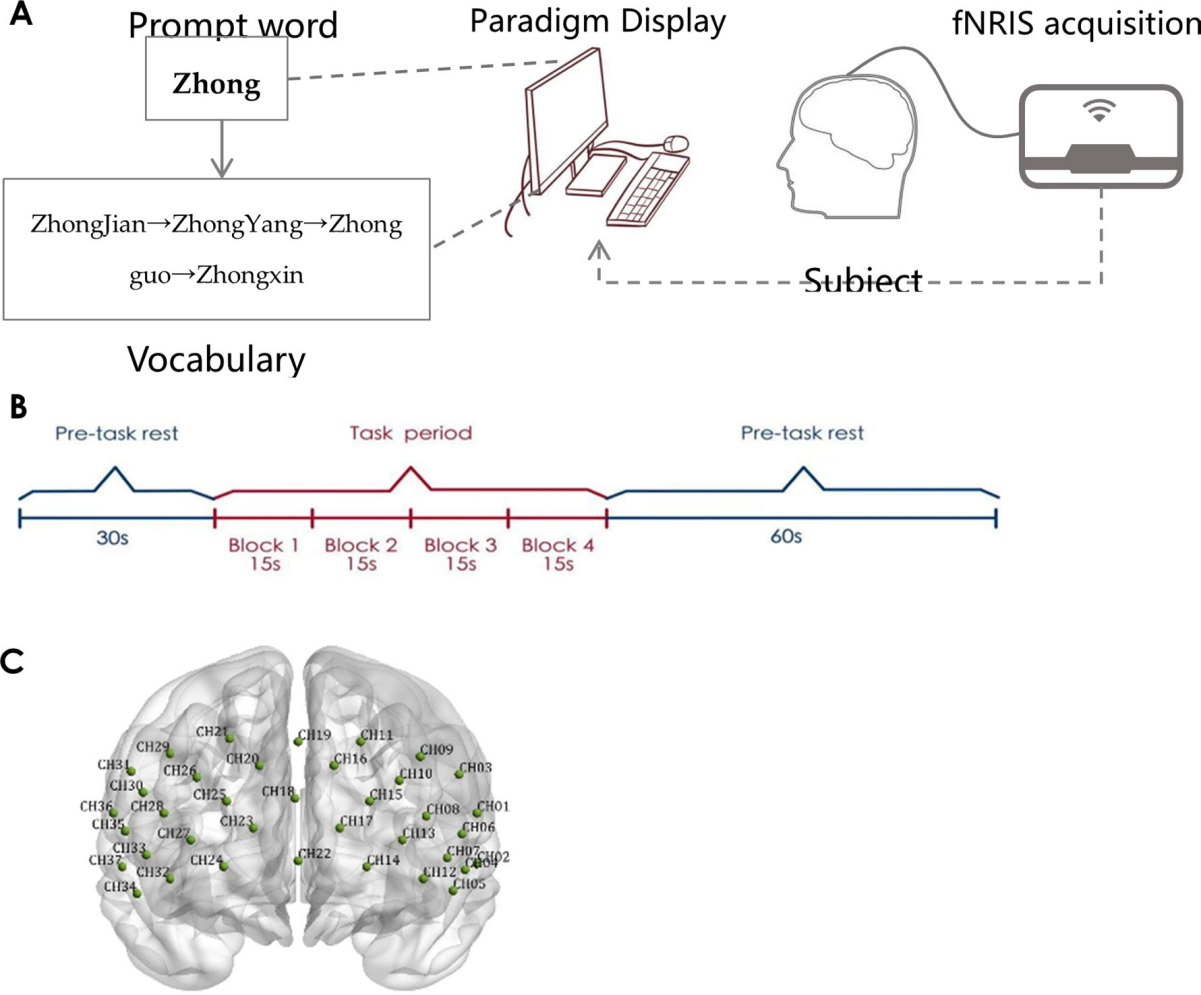
The VFT-fNIRS experiment used in this study is shown in the figure. (A) The experimental flow of this study; (B) timing diagram shown by the VFT paradigm; (C) fNIRS lead setup used in this study; the green part of the graph is the midpoint of the channel measurement. fNIRS, functional near-infrared spectroscopy; VFT, verbal fluency task.

First, the task design: the educational hierarchical VFT paradigm (table 1). The task flow was 30 s before the task→ 60 s during the task→ and 60 s after the task (figure 1A). The word frequency is classified according to education level: low-frequency words (undergraduate and above), medium-frequency words (primary school or above and below undergraduate) and high-frequency words (primary school and below), a total of four groups (one word per group, 15 s of words), and it is required to group meaningful words as much as possible (table 1). Second, the equipment parameters are set: a 37-channel fNIRS spectrometer is used to measure the light intensity signal through a dual-wavelength (695, 830 nm) laser diode according to the improved Beer-Lambert law^25^; the system consists of 12 light sources+12 detectors, with a distance of 3 cm between the light sources and detectors, and the channel is the measurement area between the two. The probe group was placed in the prefrontal lobe and bilateral temporal lobe, the lowest probe is placed according to the international 10–20 EEG electrode placement system, the optical pole 9 is used as the FPz reference point, the left and right cerebral hemispheres are symmetrically distributed along the T3–T4 line, the hind brain is fixed (figure 1B,C) and the sampling rate is set to 100 Hz.

**Table 1:** Words and frequencies used in word formation tasks

Words are frequent	Words are used for phrase tasks
Low frequency	fu, li, lian, ban
Intermediate frequency	men, kong, ku, liu
High frequency	shang, shi, shuo, jia

### Data processing and statistical analysis

The raw fNIRS data were processed in four steps: first, combined with moving SD detection (5s window) and cubic spline interpolation correction motion artefacts (threshold >0.2 OD)^26^; second, 0–0.01 Hz bandpass filtering to remove high-frequency noise (threshold signal-to-noise ratio of 20 dB)^27^; third, the change of oxyhaemoglobin concentration was calculated based on the modified Beer-Lambert law. Last, the blood oxygen concentration from 10 s before the task to 30 s after the end of the task was selected as the analysis window,^28^ and the mean difference between each channel from baseline (the last 10 s of the pretask rest time) was calculated by superimposed averages. Using IBM SPSS V.26.0 software, the demographic characteristics between the groups were first compared: the χ^2^ test (gender) and the Mann-Whitney U test (age, years of education) were used between the two groups, and the three groups were evaluated by Fisher’s exact test (gender) and Kruskal-Wallis H test (age, years of education). The statistical significance threshold was set to p<0.05. The mean value of channel HbO was compared by Mann-Whitney U test (between two groups) and Kruskal-Wallis H test (between three groups). When the Kruskal-Wallis H test indicated significant differences across three groups, pairwise comparisons were performed using Mann-Whitney U tests with Bonferroni correction, or Dunn’s post-hoc tests where appropriate.

## Result

### General data comparison results

A total of 77 patients with SLE (male: female=7:70) and 54 healthy controls (male: female=6:48) were included. Demographic characteristics (gender, age and years of education) were compared between the SLE group and the HCs group, and among the SLE subgroups (with/without depression; with/without anxiety) and HCs. There was no significant difference in age (H=1.402, p=0.161) or gender (χ^2^=0.145, p=0.703) between the SLE and HCs groups. Similarly, no significant differences in age or gender were observed among the HCs group, SLE with depression group and SLE without depression group (age: H=5.003, p=0.082; gender: p=0.083) or among the HCs group, SLE with anxiety group and SLE without anxiety group (age: H=4.108, p=0.128; gender: p=0.1337). However, years of education were significantly lower in the SLE group overall (Z=−6.457, p<0.001), in the SLE with depression subgroup (H=41.978, p<0.001) and in the SLE with anxiety subgroup (*H*=23.626, p<0.001) compared with HCs (table 2). In addition, the baseline characteristics of SLE patients included in this study were balanced and met the preset inclusion criteria. There were no significant abnormalities or exclusion indications, and all met the study enrolment requirements (table 3).

**Table 2:** General data results of HCs group and SLE group, HCs group and SLE group with or without depression group, HCs group and SLE group with or without anxiety group

	Number	Gender n (%)		Age (years)	Years of education
		Male	Female		
HCs	54	6 (11.11%)	48 (88.89%)	24 (22, 38)	19 (16, 19)
SLE	77	7 (9.09%)	70 (90.91%)	27 (11, 38)	15 (9, 16)
χ^2^/Z		0.145		1.402	−6.457
P		0.703		0.161	<0.001
HCs	46	6 (13.04%)	40 (86.96%)	26.12 (22.75, 27.75)	18.13 (16, 19)
SLE with depression group	33	0 (0%)	33 (100%)	30 (22.5, 39.5)	15 (9,15.5)
SLE without depression	44	4 (9.09%)	40 (90.91%)	27 (21, 34.75)	13.5 (9.75, 16)
χ^2^/H				5.003	41.978
P		0.083		0.082	<0.001
HCs	35	7 (20%)	28 (80%)	24 (23, 29)	19 (16, 19)
SLE with anxiety group	17	0 (0%)	17 (100%)	30 (24, 45)	15 (9, 15)
SLE without anxiety group	25	4 (16%)	21 (84%)	26 (21.5, 36.5)	15 (9, 16)
χ^2^/H				4.108	23.626
P		0.134		0.128	<0.001

The total SLE cohort comprised 76 patients who completed the full protocol; however, one additional patient with valid fNIRS data was included in the neuroimaging analysis (n=77 in tables 4 and 5). Healthy control sample sizes vary across subgroup comparisons because participants were matched to the corresponding SLE subsets for age and gender; unmatched participants were excluded to ensure balanced demographics.

fNIRS, near-infrared spectroscopy; HCs, healthy controls; SLE, systemic lupus erythematosus.

**Table 3:** Baseline data of patients with systemic lupus erythematosus

Characteristic	Value
Disease activity	
SLEDAI-2K score (n=74), median (IQR)	8.00 (4.00, 16.00)
SLE with depression group vs SLE without depression	9.00 (4.00,17.50) vs 9.00 (4.00, 16.75), p=0.92
SLE with anxiety group vs SLE without anxiety group	6.00 (4.00, 11.50) vs 10.00 (4.00, 17.25), p=0.47
Light activity (≤6), n (%)	33 (44.59)
Mild activity (6–12), n (%)	16 (21.62)
Moderate to severe activity (>12), n (%)	25 (33.78)
Clinical manifestations(n=76)	
Lupus nephritis, n (%)	42 (55.26)
Haematologic involvement, n (%)	19 (25.00)
Arthritis, n (%)	2 (2.63)
Serositis, n (%)	4 (5.26)
Gastrointestinal involvement, n (%)	5 (6.58)
Renal parameters(n=60)	
24 hours urinary microprotein>30 mg/L, n (%)	38 (63.33)
24 hours urinary total protein>0.15 g/24 hours, n (%)	44 (73.33)
Abnormal 24 hours urinary creatinine, n (%)	38 (63.33)
Hematologic abnormalities(n=74)	
Leucopenia (<4.0 or >10 ×10⁹/L), n (%)	36 (48.65)
Anaemia (Hb<110 g/L), n (%)	25 (33.78)
Thrombocytopenia (<100 ×10⁹/L), n (%)	15 (20.27)
Serological markers, n (%)	
Anti-Smith antibody (n=49) positive	19 (38.78)
Antineutrophil cytoplasmic antibody (ANCA) (n=53) positive	8 (15.09)
Anti-β2-glycoprotein I IgM (n=57) positive	7 (12.28)
Antiphospholipid antibodies (n=57) positive	5 (8.77)
Anti-cardiolipin IgG (n=57) positive	4 (7.02)
Anti-cardiolipin IgM (n=57) positive	1 (1.75)
Complement levels, median (IQR), g/L(n=73)	
C3 (normal range 0.70–1.40)	0.60 (0.37, 0.85)
C3 decreased (<0.70), n (%)	45 (61.64)
SLE with depression group vs SLE without depression	0.61 (0.39, 0.80) vs 0.51 (0.38,0.86), p=0.79
SLE with anxiety group vs SLE without anxiety group	0.64 (0.32, 0.85) vs 0.51 (0.40, 0.81), p=0.81
C4 (normal range 0.10–0.40)	0.08 (0.04, 0.14)
C4 decreased (<0.10), n (%)	44 (60.27)
SLE with depression group vs SLE without depression	0.08 (0.04, 0.13) vs 0.07 (0.04, 0.13), p=0.66
SLE with anxiety group vs SLE without anxiety group	0.04 (0.03, 0.09) vs 0.04 (0.02, 0.07), p=0.71
Inflammatory markers	
Erythrocyte sedimentation rate (ESR) (n=70), mm/h, median (IQR)	23.0 (10.0, 49.0)
ESR elevated (>20), n (%)	39 (54.93)
C reactive protein (CRP) (n=67), mg/L, median (IQR)	1.82 (0.51, 4.67)
CRP elevated (>10), n (%)	11 (16.42)
Liver and renal function(n=74), median (IQR)	
Alanine aminotransferase (ALT), U/L (normal range 7–40)	15.15 (11.85, 25.80)
Aspartate aminotransferase (AST), U/L (normal range 13–35)	19.75 (14.53, 31.60)
Serum creatinine, μmol/L (normal range 41–73)	63.35 (56.78, 71.40)
Blood urea nitrogen, mmol/L (normal range 2.6–7.5)	4.51 (3.52, 5.83)
Electrolytes (n=67), median (IQR), mmol/L	
Potassium (normal range 3.5–5.3)	3.75 (3.56, 3.98)
Sodium (normal range 137–147)	142.4 (139.8, 144.0)
Chloride (normal range 96–108)	108.1 (105.8, 110.3)
Current treatment (n=76)	
Glucocorticoids, n (%)	50 (65.79)
Cyclophosphamide	25 (32.89)
Mycophenolate mofetil, n (%)	15 (19.74)
Hydroxychloroquine, n (%)	47 (61.84)
Cyclosporine A, n (%)	7 (9.21)
Tacrolimus, n (%)	7 (9.21)
Intravenous immunoglobulin, n (%)	10 (13.16)
Belimumab, n (%)	2 (2.63)
Use glucocorticoids alone, n (%)	1 (1.32)
Glucocorticoids+immunosuppressants, n (%)	50 (65.79)
Glucocorticoids+immunosuppressants+ biological agent, n (%)	2 (2.63)

Sample sizes for individual laboratory parameters reflect missing data due to clinical non-indication or incomplete testing at the time of enrollment.

Hb, haemoglobin; SLE, systemic lupus erythematosus; SLEDAI-2K, Systemic lupus erythematosus disease activity index 2000.

### Abnormal prefrontal lobe activation pattern

**Figure 2:**
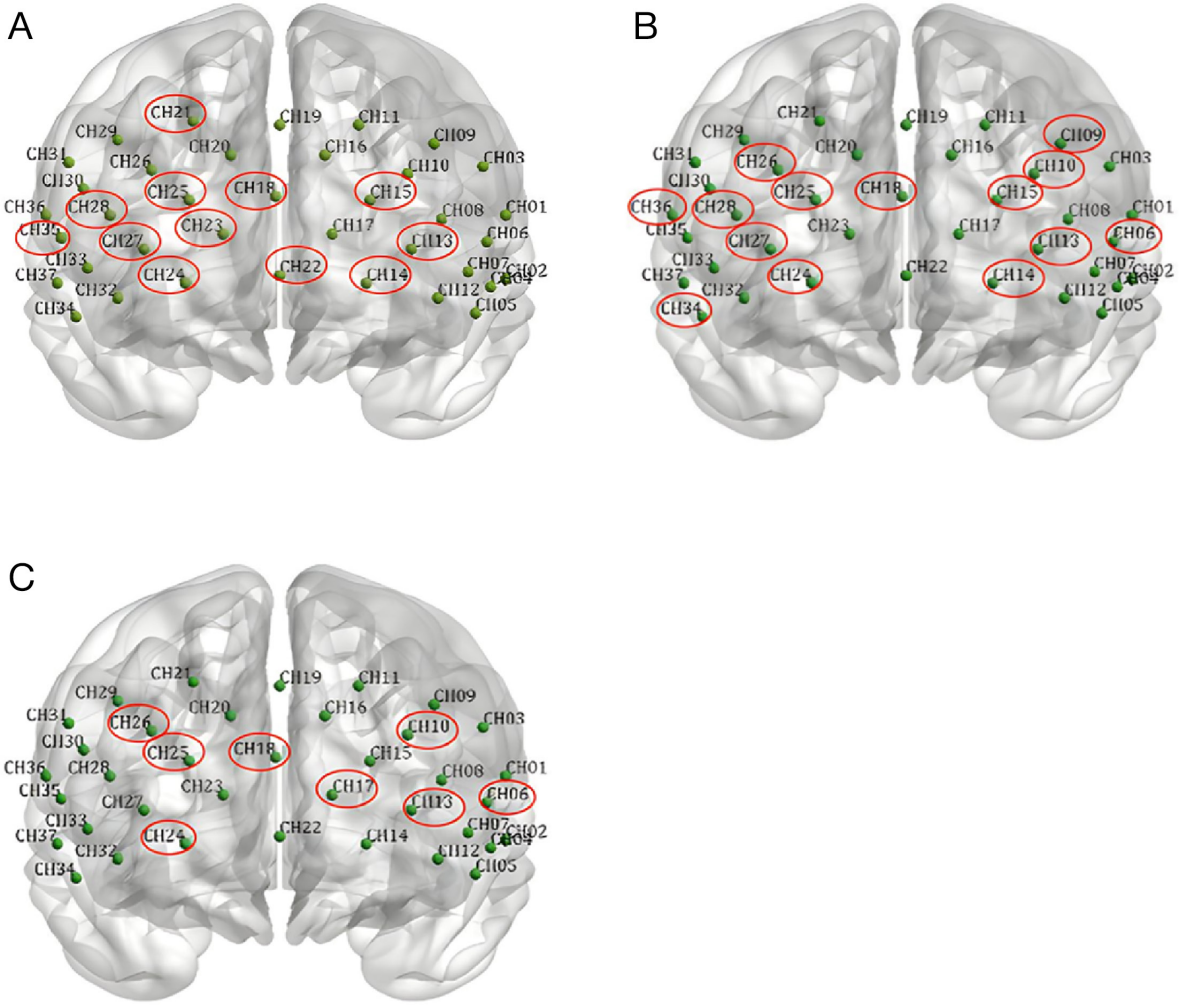
Channels showing significant group differences in HbO mean values. (**A**) Schematic diagram of 12 channels with statistically significant differences in HbO mean values between the SLE group and HCs group. (**B**) 14 channels exhibiting significant HbO mean values among the SLE with depression subgroup, SLE without depression subgroup, and HCs. (**C**) 8 channels with statistically significant differences in HbO mean values across the SLE with anxiety, SLE without anxiety and HCs group. HbO, oxyhaemoglobin; HCs, healthy controls; SLE, systemic lupus erythematosus.

The mean HbO values of the SLE group and the HCs group were statistically significant in 12 channels (CH13–15, 18, 21–25, 27, 28, 35) (p<0.05), see (table 4, figure 2A).

**Table 4:** The mean HbO values of HCs group and SLE group are statistically significant channel results

Channel	BA partition*	Brodmann name	Mean HbO (umol·cm)	Mean HbO (umol·cm)	*Z*	P value
			HCs group (n=54)	SLE group (n=77)		
CH13	BA10	Frontopolar region	1.480 (0.565, 3.354)	0.084 (0.104, 2.016)	−2.119	0.028
CH14	BA11	Orbitofrontal region	2.036 (−0.307, 3.780)	1.000 (−0.659, 2.247)	−2.295	0.022
CH15	BA10	Frontopolar region	1.624 (0.358, 3.044)	0.695 (−0.233, 1.805)	−2.430	0.015
CH18	BA10	Frontopolar region	1.578 (0.234, 2.378)	0.707 (−0.303, 1.800)	−2.362	0.018
CH21	BA9	Dorsolateral prefrontal cortex	1.267 (−0.465, 2.644)	−0.083 (−1.120, 1.408)	−2.535	0.011
CH22	BA10	Frontopolar region	1.676 (−0.615, 3.949)	0.766 (−0.943, 1.962)	−1.973	0.048
CH23	BA10	Frontopolar region	1.423 (0.667, 2.490)	0.916 (0.156, 1.938)	−2.180	0.029
CH24	BA10	Frontopolar region	1.911 (0.701, 3.650)	1.149 (0.432, 2.056)	−2.132	0.033
CH25	BA10	Frontopolar region	1.297 (0.692, 2.724)	1.010 (0.248, 1.026)	−2.309	0.021
CH27	BA10	Frontopolar region	1.771 (1.062, 3.531)	1.446 (0.539, 2.418)	−1.949	0.041
CH28	BA46	Dorsolateral prefrontal cortex	2.058 (0.848, 3.265)	1.027 (0.455, 2.101)	−2.545	0.011
CH35	BA45	pars triangularis, part of Broca’s area	1.147 (0.229, 2.191)	0.216 (-0.727, 1.529)	−2.261	0.024

HbO concentrations are expressed in μmol·cm, representing the pathlength-corrected concentration change derived from the modified Beer-Lambert law.

* BA Partition: Brodmann Partition System (see online supplemental materials for details). If the channel is associated with only one brain region, the brain region is directly labelled; if the channel is associated with two or more brain regions, the brain region with a higher degree of association is used as the marker brain region.

HbO, oxyhaemoglobin; HCs, healthy controls; SLE, systemic lupus erythematosus.

### Functional gradient characterisation of depressive symptoms

There were statistically different HbO values between the SLE with depression group and the SLE without depression group and the HCs group in 14 channels: CH6, 9, 10, 13–15, 18, 24–28, 34 and 36 (p<0.05). Moreover, the mean value of HbO in the HCs group was greater than that in the SLE group without depression, which in turn was greater than that in the SLE group with depression (table 5, figure 2B).

**Table 5:** Statistically significant channel results of HbO in HCs group and SLE with depression, SLE without depression

Channel	BA partition*	Brodmann name	Mean HbO (umol·cm)	Mean HbO (umol·cm)	Mean HbO (umol·cm)	H	P value
			HCs group (n=46)	SLE with depression group (n=33)	SLE without depression group (n=44)		
CH6	BA45	pars triangularis, part of Broca’s	1.180 (0.448, 2.256)	0.634 (0.198, 1.19)	0.531 (0.249, 1.522)	9.982	0.007
CH9	BA46	Dorsolateral prefrontal cortex	1.447 (0.614, 2.721)	0.788 (0.320, 1.4823)	0.651 (0.283, 1.418)	9.617	0.008
CH10	BA46	Dorsolateral prefrontal cortex	0.923 (0.375, 2.212)	0.388 (0.179, 0.635)	0.514 (0.296, 1.067)	10.966	0.004
CH13	BA10	Frontopolar region	0.715 (0.24 1, 1.408)	0.354 (0.203, 0.579)	0.448 (0.149, 0.987)	7.432	0.024
CH14	BA11	Orbitofrontal region	1.254 (0.585, 1.760)	0.473 (0.263, 1.204)	0.578 (0.417, 1.263)	11.338	0.003
CH15	BA10	Frontopolar region	0.607 (0.279, 1.173)	0.331 (0.153, 0.821)	0.397 (0.167, 0.647)	6.482	0.039
CH18	BA10	Frontopolar region	0.595 (0.259, 0.925)	0.264 (0.158, 0.495)	0.359 (0.146, 0.644)	7.027	0.030
CH24	BA10	Frontopolar region	0.935 (0.444, 1.370)	0.402 (0.183, 0.798)	0.436 (0.237, 0.860)	11.899	0.003
CH25	BA10	Frontopolar region	0.527 (0.257, 0.954)	0.285 (0.107, 0.465)	0.424 (0.146, 0.719)	7.572	0.023
CH26	BA46	Dorsolateral prefrontal cortex	0.754 (0.423, 1.308)	0.571 (0.117, 0.751)	0.405 (0.206, 0.788)	7.247	0.027
CH27	BA10	Frontopolar region	0.774 (0.402, 1.406)	0.438 (0.146, 0.701)	0.637 (0.209, 0.947)	163	0.006
CH28	BA46	Dorsolateral prefrontal cortex	0.752 (0.499, 1.325)	0.447 (0.205, 0.620)	0.422 (0.229, 0.947)	9.635	0.008
CH34	BA46	Dorsolateral prefrontal cortex	0.657 (0.338, 1.448)	0.333 (0.114, 0.643)	0.327 (0.139, 0.747)	12.459	0.002
CH36	BA44	pars opercularis Broca’s area	0.285 (0.185, 0.782)	0.615 (0.202, 0.977)	0.551 (0.242, 1.289)	6.968	0.031

Sample sizes after matching for gender and age: HCs (n=46), SLE with depression (n=33) and SLE without depression (n=44). Some participants were excluded during matching to ensure balanced demographics. HbO concentrations are expressed in μmol·cm.

* BA Partition: Brodmann Partition System (see online supplemental materials for details).

HbO, oxyhaemoglobin; HC, healthy control; SLE, systemic lupus erythematosus.

### Spatiotemporal response characteristics of anxiety symptoms

There were significant differences in the mean HbO values of SLE with anxiety, SLE without anxiety group and HCs group in eight channels (6, 10, 13, 17, 18, 24, 25, 26) (p<0.05). Moreover, the mean value of HbO in the HCs group was greater than that in the SLE group without anxiety and greater than that in the SLE group with anxiety. There were no statistically significant differences in the other channels (table 6, figure 2C).

**Table 6:** Statistically significant channel results of HbO in HCs group and SLE with anxiety group, SLE without anxiety

Channel	BA partition*	Brodmann name	Mean HbO (umol·cm)	Mean HbO (umol·cm)	Mean HbO (umol·cm)	H	P value
			HCs group (n=35)	SLE with anxiety group (n=17)	SLE without anxiety group (n=25)		
CH6	BA45	pars triangularis, part of Broca’s area	1.081 (0.450, 2.030)	0.417 (0.150, 1.186)	0.520 (0.228, 0.999)	8.536	0.014
CH10	BA46	Dorsolateral prefrontal cortex	0.775 (0.376, 1.340)	0.253 (0.159, 0.474)	0.704 (0.308, 1.190)	13.907	0.001
CH13	BA10	Frontopolar region	0.695 (0.276, 1.239)	0.234 (0.130, 0.396)	0.510 (0.098, 1.000)	9.519	0.009
CH17	BA10	Frontopolar region	0.876 (0.627, 1.809)	0.435 (0.186, 0.467)	0.683 (0.344, 1.337)	7.712	0.021
CH18	BA10	Frontopolar region	0.570 (0.264, 0.787)	0.236 (0.115, 0.467)	0.277 (0.120, 0.627)	7.615	0.022
CH24	BA10	Frontopolar region	0.690 (0.448, 1.162)	0.412 (0.181, 0.677)	0.421 (0.221, 0.821)	6.385	0.041
CH25	BA10	Frontopolar region	0.535 (0.254, 0.760)	0.247 (0.061, 0.418)	0.457 (0.189, 0.803)	6.250	0.044
CH26	BA46	Dorsolateral prefrontal cortex	0.746 (0.337, 1.185)	0.439 (0.101, 0.574)	0.574 (0.230, 1.07)	7.018	0.030

Sample sizes after matching for gender and age: HCs (n=35), SLE with anxiety (n=17) and SLE without anxiety (n=25). Some participants were excluded during matching to ensure balanced demographics. HbO concentrations are expressed in μmol·cm.

* BA Partition: Brodmann Partition System (see online supplemental materials for details).

HbO, oxyhaemoglobin; HCs, healthy controls; SLE, systemic lupus erythematosus.

## Discussion

### Brain network system: the regulatory role of each related brain region

In the core brain region, the Broca region (BA44/BA45) is the key centre of language production, responsible for language expression, motion programming and semantic processing. The dorsolateral prefrontal lobe (BA9/BA46) dominates executive functions, including working memory maintenance, decision-making, cognitive control and goal-oriented regulation^29^ and is central to complex task coordination and strategy selection. The frontal polar region (BA10) focuses on advanced cognitive integration, involving cross-domain information processing, innovative thinking, long-term goal planning and social cognition, helping to flexibly transform cognition in complex scenarios. The premotor cortex (BA6) is responsible for the synergistic integration of motor planning and language-action, providing preregulation of language expression and somatic movement. The orbitofrontal lobe (BA11) focuses on emotional decision-making and reward processing, participates in emotional value evaluation and preference formation and coordinates the synergistic operation of emotions and cognition. The temporal polar region (BA38) is related to memory extraction and emotional language integration and assists episodic memory arousal and emotional language processing. The frontal orbital region (BA47) is mainly involved in sensory integration functions, including decision-making emotion and motivational control, cognitive flexibility and social behaviour^30 31^ and is involved in some executive functions, such as selecting, comparing and judging stimuli in short-term and long-term memory.^32^ These brain regions form a functional network of language expression, cognitive regulation, emotional integration and action coordination through multichannel cross-coverage, providing core neural foundation support for fNIR research.

### NPSLE dynamic biological indicators system construction

Changes in the frontal cortex are associated with impaired performance of specific cognitive tasks,^33^ and the prefrontal cortex is responsible for cognitive activities including memory, reasoning, execution and decision-making. The regions of prefrontal dysfunction (F3/Fp1) found in this study highly overlap spatially with the regions of hypometabolism in the frontal lobe reported in previous PET studies,^34 35^ suggesting that fNIRS may have captured early changes in oxygen metabolism in SLE neurodegeneration. The results of this study confirmed that the peak concentration of oxyhaemoglobin and the mean concentration of oxyhaemoglobin on multiple channels covering the dorsolateral and ventrolateral of the prefrontal lobe were significantly lower than those of the healthy control group. These channels are mainly located in areas BA9 and 46, which are known to be closely related to executive function, working memory and language production. This finding strongly supports our overarching hypothesis that there is a pervasive underactivation of the prefrontal cortex in patients with SLE.

Patients in the group with depression showed a more severe reduction in oxyhaemoglobin concentrations on a specific series of channels (14 channels) compared with patients without depression. These channels are spatially concentrated in the DLPFC and bilateral frontal regions. Correspondingly, patients in the symptom group with anxiety symptoms showed reduced specific activation on another group of channels adjacent to but partially non-overlapping with the DLPFC, with a total of eight channels reaching a significant level. Of particular note is that a channel (CH10) located in the right DLPFC showed the strongest effect in the anxiety symptom group, but the difference was not significant in the depressive symptom group, suggesting that anxiety symptoms may be more specifically associated with the function of the right prefrontal lobe. Although depressive and anxiety symptoms often coexist and there is partial overlap in the brain regions with related functional abnormalities, the current differences in spatial distribution patterns still initially suggest that depressive and anxiety symptoms may correspond to partially separable prefrontal cortex dysfunction patterns in patients with SLE. At the same time, the results of this study showed that the concentration of oxyhaemoglobin in the dorsal prefrontal cortex (BA9 and BA46 regions) of patients with SLE was lower than that of the healthy control group (q<0.05), indicating that there was insufficient oxygen supply and blood flow perfusion in this brain region, and the energy metabolism and basic physiological activities of nerve cells were impaired. These pathological changes can induce a chronic inflammatory response, which further reduces the efficiency of neurovascular coupling by disrupting the integrity of the blood–brain barrier,^36 37^ which may eventually lead to neuropsychiatric symptoms in patients with SLE. These data indicate that prefrontal blood oxygen oscillation disturbances are associated with depressive and anxiety symptoms in SLE and occur during cognitive task engagement; however, without formal neuropsychological testing, they cannot be interpreted as direct biomarkers of objective cognitive impairment. In addition, specific regions in the study can be used as key targets for neuropsychiatric symptoms in patients with SLE, providing a certain basis for the development of neuromodulation therapies such as transcranial magnetic stimulation.

### Differences in oxygen activation patterns between SLE and primary mental disorders

Compared with major depressive disorder (MDD), in previous fNIRS-VFT studies, Wu H *et al* found that the blood oxygen activation in regions such as the right DLPFC and bilateral frontopolar cortex (FPC) of MDD patients was significantly lower than that of healthy controls.^38^ Our SLE with depression subgroup showed a similar distributed reduction across 14 channels covering BA10/BA46. However, in primary MDD, the spatial extent is more often prefrontal than usually reported,^39 40^ indicating that SLE-related depressive symptoms may involve different frontopolar microvascular dysfunction, superimposed on autoimmune-mediated pathology. In the study of primary anxiety disorders, fNIRS studies on generalised anxiety disorder and panic disorder described the overactivation of the right DLPFC and ventral prefrontal cortex during cognitive challenges, which was interpreted as compensatory over-recruitment.^41^ In contrast, our SLE with anxiety subgroup showed specific hypoxia in the right DLPFC (CH10) and left frontopolar region, indicating hypofunction rather than hyperactivity of the prefrontal lobe. This difference supports the hypothesis that anxiety in patients with SLE may be driven by autoimmune-mediated hypoperfusion/neurovascular decoupling, rather than the functional hyperactivity seen in primary anxiety disorders. In terms of the research on dementia/cognitive impairment, although our cohort did not include dementia patients (mean age 27 years), we cited PET/fMRI studies on SLE-related cognitive dysfunction, which showed that the frontal lobe hypometabolic pattern spatially overlapped with our fNIRS hypoxia clusters (F3/Fp1).^11 12^ This comparison suggests that the prefrontal oxygen oscillation defects detected by fNIRS may represent an early functional correlate of metabolic disruption in advanced neuropsychiatric lupus. We clearly point out that the symptom-specific spatial gradients observed in our SLE cohort (anxiety concentrated in the right BA46; depression more diffusely distributed in BA10/BA46) are different from the relatively uniform prefrontal lobe dysfunction reported in primary mental illnesses. This supports our central argument that prefrontal HbO oscillations in SLE represent a disease-specific and dynamically relevant biomarker, rather than a non-specific epiphenomenon of emotional symptoms. However, the absence of disease-specific (psychiatric) control groups does limit definitive transdiagnostic comparisons to some extent. Future multicentre studies by our group will include cohorts with primary MDD and generalised anxiety disorder to prospectively validate these distinct patterns.

### Research limitations and future prospects

The aetiology of NPSLE is multifactorial and may include cerebrovascular lesions, direct attack by autoantibodies on nerve cells, disruption of the blood–brain barrier and systemic inflammatory factors affecting the central nervous system through neuroimmune pathways. The weakened response of prefrontal oxyhaemoglobin found in this study may be the embodiment of the eventual convergence of these underlying pathophysiological processes at the functional level of the cortex. Therefore, the signals measured by fNIR spectroscopy may integrate information on neural activity, vascular reactivity and metabolic status. Compared with fMRI and PET, fNIRS is particularly suitable for outpatient due to its portability (device weight <2 kg), high temporal resolution (sampling rate 10.2 Hz) and antimotion artefact properties (head movement tolerance >5 mm), which has unique advantages in outpatient screening and long-term monitoring.^15 42^ Therefore, future studies need to combine fNIR spectroscopy indicators with more direct immune-inflammatory markers (such as peripheral blood cytokine levels) and cerebrovascular function parameters to more deeply dissect the biological mechanisms behind them. The application of functional near-infrared spectroscopy technology to the assessment of NPSLE has shown great potential. Compared with fMRI, fNIRS is more clinically feasible and convenient for dynamic monitoring in outpatient settings. Our study shows that fNIR spectroscopy measurements based on VFT are able to sensitively detect changes in brain function associated with clinical symptoms. This lays the foundation for possible future application scenarios: first, as an auxiliary diagnostic tool to help identify whether neuropsychiatric symptoms that are difficult to characterise are related to SLE itself; second, as relevant biological indicators for efficacy evaluation, reflecting the effect of drug or non-drug intervention on brain function; third, as a predictor, identifying those patients with insufficient prefrontal function reserve and are at high risk of developing severe neuropsychiatric symptoms.

However, there are limitations to this study: first, the cross-sectional design fails to establish a causal relationship between prefrontal dysfunction and neuropsychiatric symptoms, both of which may be a common outcome of the SLE disease process, or the relationship is bidirectional. Second, although we have made efforts to control confounding factors such as educational level, residual confounding is still difficult to completely avoid. For example, we were unable to comprehensively assess the cognitive reserve and vascular risk factors of all participants. Moreover, the sample size of this study is relatively limited, and there is a gender-biased distribution (with women accounting for >90%). In the future, validation is needed in a large-scale, multi-centre cohort. Third, our assessment of the autoantibody levels in the SLE group is also incomplete. Based on the above research limitations, future studies should also consider combining a comprehensive serological test panel for NPSLE, including anti-double-stranded DNA antibodies, antiribosomal P antibodies, anti-N-methyl-D-aspartate receptor NR2 subunit antibodies and antineuronal antibodies, with fNIRS indicators, so as to clarify whether a specific autoantibody profile is the core driving factor, leading to abnormal prefrontal blood oxygen oscillations. Fourth, the exclusion of patients requiring high-dose glucocorticoids therapy (>10 mg/day prednisone-equivalent) limits generalisability to severely active SLE cohorts. However, this criterion was necessary to minimise the confounding effects of exogenous corticosteroids on prefrontal haemodynamics and neuropsychiatric symptoms. Future studies should stratify analyses by cumulative GC exposure or include a high-dose GC subgroup. Finally, fNIRS mainly detects the activity of superficial cortical regions, which cannot be directly assessed for the function of deep brain structures (eg, amygdala, hippocampus), which are also crucial in emotional processing.

## Conclusion

This study successfully delineated the specific association between neuropsychiatric symptoms and functional activity of the prefrontal cortex in patients with SLE through fNIRS imaging techniques quantified by the educational hierarchical VFT paradigm. Our results confirm that task-induced oxyhaemoglobin response abnormalities are common in patients with SLE, and that decreased HbO responses in SLE patients with anxiety are specific to the prefrontal subregion (BA46 dorsolateral prefrontal region), while SLE patients with depression show a more distributed pattern. These findings support the hypothesis that prefrontal oxygen oscillation disturbances are dynamic biological indicators associated with depressive and anxiety symptoms in NPSLE during cognitive task performance, providing novel insights into the neurobiological basis of mood disturbances in SLE.

## Supplementary material

10.1136/lupus-2026-002009 online supplemental file 1

## Data Availability

Data are available upon reasonable request.
