# Supplementary material for: Quantitative mapping of prefrontal oxygenation dysfunction and neuropsychiatric symptoms in patients with systemic lupus erythematosus: a multichannel functional near-infrared spectroscopy study
Source: Lupus Sci Med. 2026 Jul 1;13(2):e002009. doi: 10.1136/lupus-2026-002009 (PMC13331049; doi:10.1136/lupus-2026-002009)
Supplement: online supplemental file 1 [file lupus-13-2-s001.docx]

Table of fNIR 37 channels - BA partition proportion and function classification

| Channel | Corresponding Brodmann Partition (BA) and Percentage | Classification of core brain area functions |
| --- | --- | --- |
| CH01 | BA6: 0.18893；  BA44: 0.557；  BA45: 0.25407 | Premotor cortex + Broca's area (verbal motor control + complex motor planning) |
| CH02 | BA44: 0.70035；  BA45: 0.29965 | Broca Area Core (Language Production and Expression Regulation) |
| CH03 | BA38: 0.25552；  BA45: 0.68454；  BA47: 0.0031546；  BA48: 0.056782 | Temporal polar region + Broca's area (emotional language integration and episodic memory extraction) |
| CH04 | BA45: 0.27083；  BA46: 0.44444；  BA47: 0.28472 | Dorsolateral prefrontal lobe + Broca's area (executive function and linguistic semantic processing) |
| CH05 | BA45: 1.0 | Broca area (the core execution area of language generation) |
| CH06 | BA45: 0.98491；  BA46: 0.015094 | Broca's area is dominant (language function is mainly and micro-executive function is assisted) |
| CH07 | BA45: 0.35612；  BA46: 0.64388 | Dorserolateral prefrontal lobe dominance (executive function and language-cognitive coordination) |
| CH08 | BA10: 0.0039683；  BA45: 0.17857；  BA46: 0.81746 | Dorserolateral prefrontal lobe dominance (executive function core and frontal pole cognitive aids) |
| CH09 | BA9: 0.31797；  BA45: 0.2765；  BA46: 0.40553 | Dorsolateral prefrontal lobe (working memory maintenance and verbal decision integration) |
| CH10 | BA9: 0.017467；  BA45: 0.0087336；  BA46: 0.9738 | Dorsolateral prefrontal core (high proportion of executive function regulatory area) |
| CH11 | BA9: 0.96818；  BA46: 0.031818 | Dorserolateral prefrontal lobe (executive function and cognitive control core) |
| CH12 | BA10: 0.43969；  BA11: 0.0038911；  BA46: 0.50973；  BA47: 0.046693 | Frontal pole + dorsolateral prefrontal lobe (advanced decision-making and goal-directed executive functions) |
| CH13 | BA10: 0.8；  BA11: 0.011111；  BA46: 0.18889 | Frontal polar dominance (cross-task cognitive transformation and value judgment) |
| CH14 | BA10: 0.48837；  BA11: 0.51163 | Frontal pole + orbitofrontal lobe (emotional decision-making and reward-expectant processing) |
| CH15 | BA10: 0.80545；  BA46: 0.19455 | Frontal polar dominance (long-term goal planning and cognitive strategy selection) |
| CH16 | BA9: 0.54406；  BA10: 0.43678；  BA46: 0.019157 | Dorserolateral prefrontal lobe + frontal pole (working memory and decision assessment synergy) |
| CH17 | BA10: 1.0 | Frontal polar region (advanced cognitive integration and innovative thinking regulation) |
| CH18 | BA10: 1.0 | Frontal polar region (advanced cognitive integration and innovative thinking regulation) |
| CH19 | BA9: 1.0 | Dorsolateral prefrontal lobe (working memory encoding and executive function regulation) |
| CH20 | BA9: 0.51321；  BA10: 0.48679 | Dorsolateral prefrontal lobe + frontal pole (working memory and decision making synergy) |
| CH21 | BA9: 0.99115；  BA46: 0.0088496 | Dorserolateral prefrontal lobe dominance (high-purity executive function and cognitive control) |
| CH22 | BA10: 1.0 | Frontal polar region (strategic coordination of complex cognitive tasks) |
| CH23 | BA10: 1.0 | Frontal polar region (social cognition and psychological theory processing) |
| CH24 | BA10: 0.50658；  BA11: 0.49342 | Frontal pole + orbitofrontal lobe (emotional value assessment and decision-making preference formation) |
| CH25 | BA10: 0.90076；  BA46: 0.099237 | Frontal polar region dominance (cognitive goal prioritization + executive monitoring) |
| CH26 | BA9: 0.10502；  BA46: 0.89498 | Dorsolateral prefrontal core (precise regulation of executive function) |
| CH27 | BA10: 0.90262；  BA11: 0.041199；  BA46: 0.05618 | Frontal polar region dominance (emotion - cognitive integration and executive function fine-tuning) |
| CH28 | BA10: 0.016327；  BA45: 0.15102；  BA46: 0.83265 | Dorserolateral prefrontal lobe dominance (executive function + verbal cognitive aid) |
| CH29 | BA9: 0.39535；  BA45: 0.24186；  BA46: 0.36279 | Dorserolateral prefrontal lobe (working memory and collaborative regulation of language decisions) |
| CH30 | BA45: 0.97719；  BA46: 0.022814 | Broca's area dominance (language production and microexecutive function assistance) |
| CH31 | BA44: 0.62633；  BA45: 0.37367 | Broca's area dominance (language production and microexecutive function assistance) |
| CH32 | BA10: 0.44269；  BA11: 0.027668；  BA46: 0.45059；  BA47: 0.079051 | Frontal pole and dorsolateral prefrontal lobe (decision making + executive function + emotional integration) |
| CH33 | BA45: 0.22662；  BA46: 0.77338 | Dorserolateral prefrontal lobe dominance (executive function and language-cognitive coordination) |
| CH34 | BA45: 0.19795；  BA46: 0.48464；  BA47: 0.31741 | Dorsolateral prefrontal lobe + inferior frontal gyrus (executive function and verbal semantic processing) |
| CH35 | BA45: 1.0 | Broca area (the core execution area of language generation) |
| CH36 | BA6: 0.17857；  BA44: 0.50974；  BA45: 0.31169 | Premotor cortex + Broca's area (motor - language integration regulation) |
| CH37 | BA38: 0.1885；  BA45: 0.77316；  BA48: 0.038339 | Temporal polar region + Broca's region (emotional language processing + memory extraction synergy) |
